# Comparative Study of the Efficacy of Ranolazine as Add-On Therapy With Metformin Versus Metformin Monotherapy on Glycaemic Control in Patients of Type 2 Diabetes Mellitus

**DOI:** 10.7759/cureus.101227

**Published:** 2026-01-10

**Authors:** Nidhi Kumari, Amrendra K Arya, Ved Prakash, Saajid Hameed, Hitesh Mishra, Harihar Dikshit

**Affiliations:** 1 Department of Pharmacology, Indira Gandhi Institute of Medical Sciences, Patna, IND; 2 Department of Endocrinology, Indira Gandhi Institute of Medical Sciences, Patna, IND

**Keywords:** adjunct therapy, hba1c, metformin, ranolazine, type 2 diabetes mellitus

## Abstract

Background: Type 2 diabetes mellitus (T2DM), being a progressive metabolic disorder, often needs combination therapy for optimal glycaemic control. Metformin is the standard first-line agent, but its efficacy may diminish over time. Ranolazine, an antianginal drug with emerging antihyperglycaemic properties, has shown promise in improving glucose regulation. This study evaluated the efficacy of ranolazine as an add-on therapy to metformin compared with metformin monotherapy.

Methods: A quasi-experimental comparative study was conducted in a tertiary care hospital in eastern India. Adults aged 18-75 years with newly diagnosed or metformin-stabilised T2DM (HbA1c 6.5-9%) were enrolled. Group M received metformin 1000 mg once daily, while Group M+R received metformin 1000 mg once daily plus ranolazine (500 mg twice daily, up-titrated to 1000 mg twice daily). Follow-up of patients was conducted for six months, with assessments at baseline, three months, and six months. The primary endpoint was the mean reduction in HbA1c at three months, while secondary endpoints included changes in fasting blood glucose (FBG), post-prandial blood glucose (PPBG), and the need for rescue medication.

Results: Of 120 randomised patients, 54 in Group M and 58 in Group M+R completed the study. At three months, the combination group achieved a significantly greater HbA1c reduction (mean difference 0.25%, p=0.022), with sustained benefit at six months (mean difference 0.23%, p=0.037). FBG and PPBG were significantly lower in the combination group at both three and six months. Regression analysis identified longer diabetes duration, higher LDL, and elevated baseline HbA1c as predictors of HbA1c change. No increased risk of hypoglycaemia was observed.

Conclusion: Ranolazine, when added to metformin, provides incremental and statistically significant improvements in glycaemic control, supporting its potential role as an adjunct therapy in T2DM. Larger, longer-term studies are warranted to confirm durability and cardiovascular benefits.

## Introduction

Type 2 diabetes mellitus (T2DM) is a chronic metabolic disorder characterised by insulin resistance and relative insulin deficiency, resulting in persistent hyperglycaemia [1]. Epidemiologically, T2DM represents a major global health challenge [2]. Its prevalence continues to rise, particularly in low- and middle-income countries, driven by urbanisation, ageing populations, and increasing obesity rates, with projections exceeding 700 million cases by 2045 [3].

The clinical burden of T2DM is profound, encompassing both microvascular complications (such as retinopathy, nephropathy, and neuropathy) and macrovascular complications, including cardiovascular disease, stroke, and peripheral artery disease, which remain leading causes of morbidity and mortality [4,5]. Given this dual spectrum of complications, effective management requires not only glycaemic control but also strategies that mitigate cardiovascular risk.

Metformin remains the cornerstone of pharmacological therapy for T2DM, recommended as first-line treatment by major guidelines due to its efficacy, safety, and cost-effectiveness [6,7]. Its mechanism of action, that is, reducing hepatic glucose production, enhancing insulin sensitivity, and improving peripheral glucose uptake, makes it a reliable agent for lowering blood glucose without significant hypoglycaemia [8,9]. However, metformin monotherapy has limitations. Gastrointestinal intolerance leads to discontinuation in up to 5-10% of patients [10,11], and its effectiveness may wane over time as beta-cell function declines. Moreover, metformin does not fully address progressive insulin deficiency or severe insulin resistance, underscoring the need for combination therapy to achieve durable glycaemic control and address the multifactorial pathophysiology of T2DM [12].

Ranolazine, an anti-anginal agent that inhibits late sodium currents, has emerged as a potential adjunctive therapy in T2DM. By reducing intracellular sodium (Na⁺) and calcium (Ca²⁺) overload, ranolazine may protect pancreatic beta cells from glucotoxicity- and lipotoxicity-induced dysfunction, thereby supporting insulin secretion and glucose homeostasis [13]. Clinical studies suggest that ranolazine monotherapy can lower HbA1c by approximately 0.56% over 24 weeks and modestly improve insulin resistance (Homeostatic Model Assessment of Insulin Resistance, that is, HOMA-IR reduction from 3.01 to 2.8) [14,15]. Beyond glycaemic effects, ranolazine has demonstrated cardiovascular benefits, including improvements in diastolic function, regional filling rates, and reduced pulmonary capillary wedge pressure in patients with heart failure with preserved ejection fraction (HFpEF) [16-18].

These findings highlight ranolazine’s dual potential: improving glycaemic control while simultaneously addressing cardiovascular dysfunction, a critical consideration given the high burden of cardiovascular disease in T2DM. However, current evidence is limited by small sample sizes and short study durations, particularly in combination therapy settings.

We therefore hypothesise that ranolazine, when combined with metformin, will provide superior glycaemic control compared to metformin monotherapy, through complementary effects on insulin sensitivity, beta-cell function, and cardiovascular parameters. The objective of this comparative study is to evaluate the efficacy of ranolazine as an add-on therapy to metformin versus metformin alone, measured by changes in HbA1c, fasting plasma glucose (FBG), and post-prandial blood glucose (PPBG) over a 24-week period in patients with T2DM.

## Materials and methods

This quasi-experimental comparative study was undertaken through a collaborative effort between the Departments of Endocrinology and Pharmacology at a tertiary care hospital in eastern India from July 2021 to June 2022. The study protocol received prior approval (092/IEC/IGIMS/2019) from the Institutional Ethics Committee, Indira Gandhi Institute of Medical Sciences (IGIMS), Patna, ensuring adherence to ethical standards and regulatory requirements. Written informed consent was obtained from all participants before enrollment, in accordance with the principles of the Declaration of Helsinki.

Eligibility criteria

Adults aged 18-75 years with newly diagnosed T2DM or those stabilised on metformin monotherapy, having HbA1c levels between 6.5-9% and FBG between 126-240 mg/dl, were eligible for inclusion in the study. Patients were excluded if they had acute or chronic renal insufficiency, hepatic dysfunction, type 1 or secondary diabetes, were receiving other oral hypoglycaemic agents or insulin, or had experienced recent cardiovascular events within the preceding three months. Additional exclusion criteria comprised uncontrolled hypertension (>160/100 mmHg), prolonged QT interval (>500 ms), recent bariatric surgery or significant weight change within two months, and pregnancy or lactation.

Sample size

Based on an anticipated mean HbA1c reduction of 0.7 ± 0.1% with metformin monotherapy and 0.8% with metformin plus ranolazine, a minimum of 104 patients was needed to achieve 95% power at α = 0.05. Ultimately, 54 patients in the metformin group (Group M) and 58 patients in the metformin plus ranolazine group (Group M+R) completed the study per protocol.

Methodology 

Patients receiving metformin 1000 mg once daily were taken in Group M. Patients receiving metformin 1000 mg once daily plus ranolazine were enrolled in Group M+R. Ranolazine was initiated at 500 mg BD (twice daily) and then up-titrated to 1000 mg BD after seven days. Compliance was monitored using pill counts.

Patients were assessed at baseline, three months, and six months, with the primary endpoint defined as the mean reduction in HbA1c at three months. Secondary endpoints included changes in FBG and PPBG at three and six months, the need for rescue medication, and the overall change in HbA1c from baseline to six months. Laboratory evaluations were performed using standardised methods: FBG was measured by the glucose-oxidase peroxidase technique on the Beckman Coulter AU700 analyser (Brea, CA, USA), while HbA1c was determined using the Tosoh HLC-723 G8 ion-exchange high-performance liquid chromatography (HPLC) system (Tosoh, Tokyo, Japan). Baseline assessments also included lipid profile measurements and blood pressure recording.

Statistical analysis

Baseline characteristics were compared between Group M versus Group M+R using unpaired t-tests for normally distributed continuous variables (age, duration of diabetes, BMI, HbA1c, FBG, SBP or systolic blood pressure, DBP or diastolic blood pressure, total cholesterol, HDL or high-density lipoprotein) and the Mann-Whitney U test for non-normally distributed continuous variables (triglycerides, LDL or low-density lipoprotein, PPBG). Categorical variables (gender distribution) were compared using the Chi-square test or Fisher's exact test. Changes in HbA1c, FBG, and PPBG between groups at follow-up were analysed using unpaired t-tests with 95% confidence intervals. For variables not normally distributed at follow-up, non-parametric tests were applied as appropriate. Multivariable linear regression analysis was conducted to analyse other predictors of HbA1c change, including demographic, clinical, and treatment-related variables. Variables entered into the model were: age, gender, diabetes duration, BMI, baseline HbA1c, SBP, LDL level, and treatment group. A p-value <0.05 was the threshold for statistical significance.

## Results

**Figure 1:**
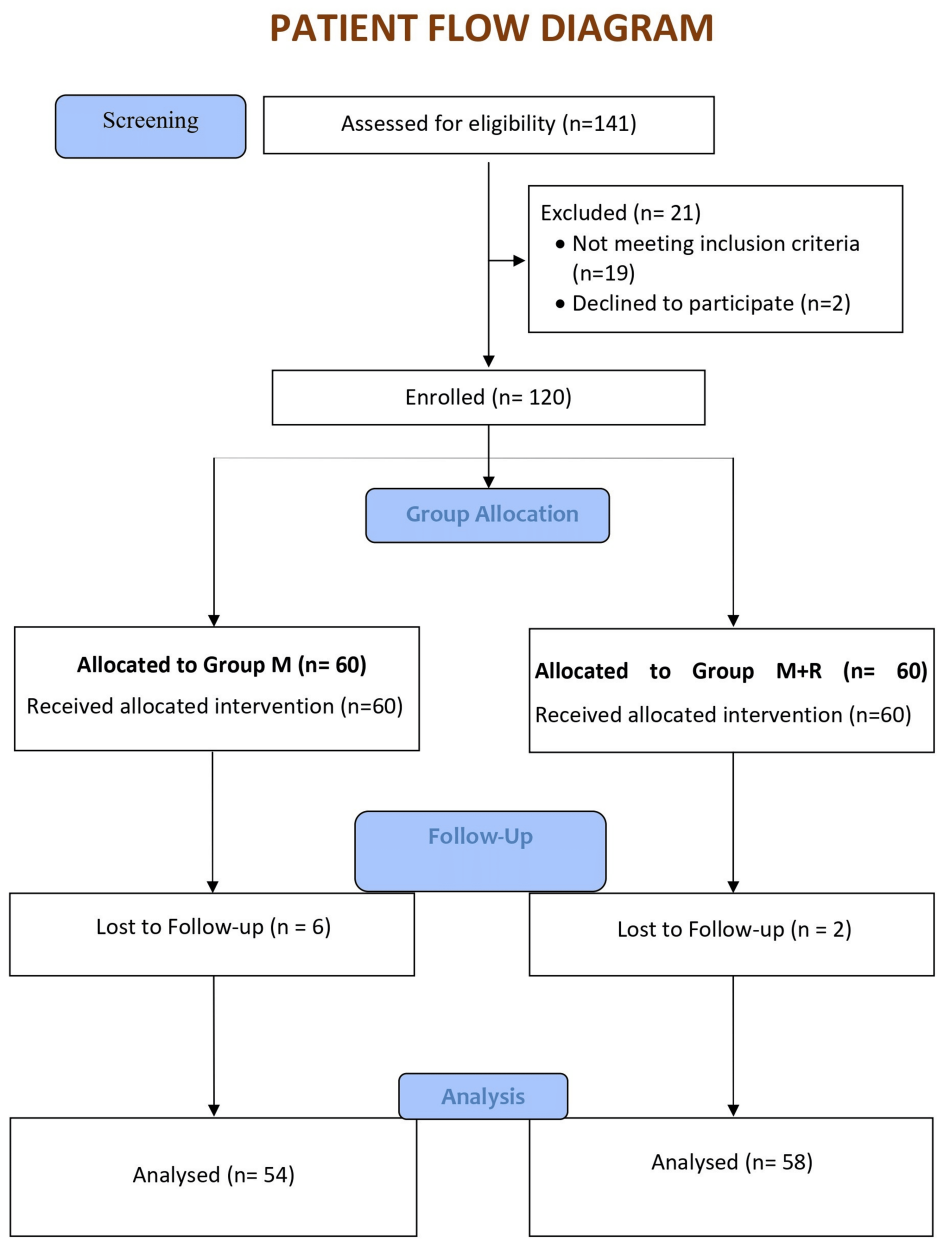
Patient flow diagram Group M: metformin group; Group M+R: metformin + ranolazine group

After follow-ups, we found that six patients from Group M and two patients from Group M+R had to be withdrawn from the study as their requirement for the addition of another hypoglycaemic drug was there. So, only 54 patients in group M and 58 patients in group M+R completed the study as per protocol (Figure 1).

Both groups had similar mean ages (52.43 in Group M vs. 51.17 years in Group M+R, p=0.373). There were 34/54 (62.96%) male subjects in Group M as compared to 20/54 (37.04%) female subjects, whereas there were 36/58 (62.07%) male subjects in Group M+R as compared to 22/58 (37.83%) female subjects (p=0.92). The median duration of diabetes was slightly longer in the metformin group (3.8 vs. 3.4 years, p=0.20), while BMI values were modestly higher in the combination group (26.8 vs. 25.8 kg/m², p=0.53). Glycaemic indices, including HbA1c (8.78% vs. 8.83%, p=0.761), FBG (167.4 vs. 166.9 mg/dl, p=0.168), and PPBG (273 vs. 275 mg/dl, p=0.373), were nearly identical. Blood pressure readings (SBP 155.5 vs. 155.2 mmHg, p=0.209; DBP 90.8 vs. 94.7 mmHg, p=0.106) and lipid profiles (total cholesterol 203.9 vs. 194.2 mg/dl, p=0.540; triglycerides 226 vs. 225 mg/dl, p=0.448; HDL 38.9 vs. 40.6 mg/dl, p=0.233; LDL 120 vs. 107 mg/dl, p=0.312) also showed no significant variation. Overall, the two groups were well matched at baseline, ensuring comparability for subsequent outcome analyses (Tables 1, 2). 

**Table 1:** Comparison of normally distributed baseline demographic and clinical variables SD: standard deviation; n: number of patients; N: total number of patients in group; FBG: fasting blood glucose; SBP: systolic blood pressure; DBP: diastolic blood pressure; HDL: high-density lipoprotein

Parameters	Metformin group (N=54)	Metformin and ranolazine group (N=58)	p-value (unpaired t-test)
Age in years, Mean ± SD	52.43 ± 10.59	51.17 ± 9.87	0.373
Body Mass Index (BMI) in kg/m^2^, Mean ± SD	25.83 ± 1.72	26.81 ± 3.58	0.53
HbA1c in %, Mean ± SD	8.779 ± 0.53	8.834 ± 0.60	0.761
FBG in mg/dl, Mean ± SD	167.388 ± 9.66	166.862 ± 10.30	0.168
SBP in mmHg, Mean ± SD	155.463 ± 9.82	155.172 ± 12.21	0.209
DBP in mmHg, Mean ± SD	90.833 ± 8.88	94.655 ± 9.40	0.106
Total cholesterol in mg/dl, Mean ± SD	203.92 ± 18.47	194.15 ± 23.73	0.540
HDL in mg/dl, Mean ± SD	38.92 ± 7.00	40.55 ± 6.12	0.233

**Table 2:** Comparison of non-normally distributed baseline demographic and clinical variables IQR: inter-quartile range; PPBG: post-prandial blood glucose; LDL: low-density lipoprotein

Parameters	Metformin group (n=54)	Metformin and ranolazine group (n=58)	p-value (Mann-Whitney U-test)
Duration of diabetes in years, Median (IQR)	3.8 (2.7 – 5.0)	3.4 (2.2 – 5.0)	0.20
PPBG in mg/dl, Median (IQR)	273 (174 – 373)	275 (187 – 342)	0.373
Triglyceride in mg/dl, Median (IQR)	226 (170 – 282)	225 (172 – 278)	0.448
LDL in mg/dl, Median (IQR)	120 (93 – 148)	107 (71 – 134)	0.312

At three months, the combination group showed a significantly greater reduction in HbA1c (8.24% vs. 8.49%, mean difference or MD: 0.25%, 95% CI: 0.037-0.465, p=0.022), and this benefit persisted at six months (7.73% vs. 7.96%, MD 0.23%, 95% CI: 0.014-0.438, p=0.037). Although the absolute change from baseline to six months was numerically larger in the combination group (1.10% vs. 0.82%), it was not significant (MD -0.28%, 95% CI: -0.583 to 0.021, p=0.068). Overall, these findings suggest that adding ranolazine to metformin provides an incremental and statistically significant improvement in glycaemic control at interim time points, with a trend toward greater long-term HbA1c reduction that narrowly missed conventional significance thresholds (Table 3).

**Table 3:** Mean HbA1c level at baseline, three months and six months after follow-up CI: confidence interval; data represented as Mean ± SD; M: metformin group; M+R: metformin + ranolazine group; * statistically significant

Variables	Metformin group (n=54)	Metformin and ranolazine group (n=58)	Difference: M – M+R (95% CI)	p-value (Unpaired t-test)
Baseline	8.779 ± 0.53	8.834 ± 0.60	-0.05500 (-0.2676 to 0.1576)	0.373
3 months	8.490 ± 0.64	8.239 ± 0.50	0.2510 (0.03673 to 0.4653)	0.022*
6 months	7.958 ± 0.62	7.732 ± 0.51	0.2260 (0.01401 to 0.4380)	0.037*
Change from baseline to 6 months	0.821 ± 0.82	1.102 ± 0.79	-0.2810 (-0.5825 to 0.02053)	0.0675

**Figure 2:**
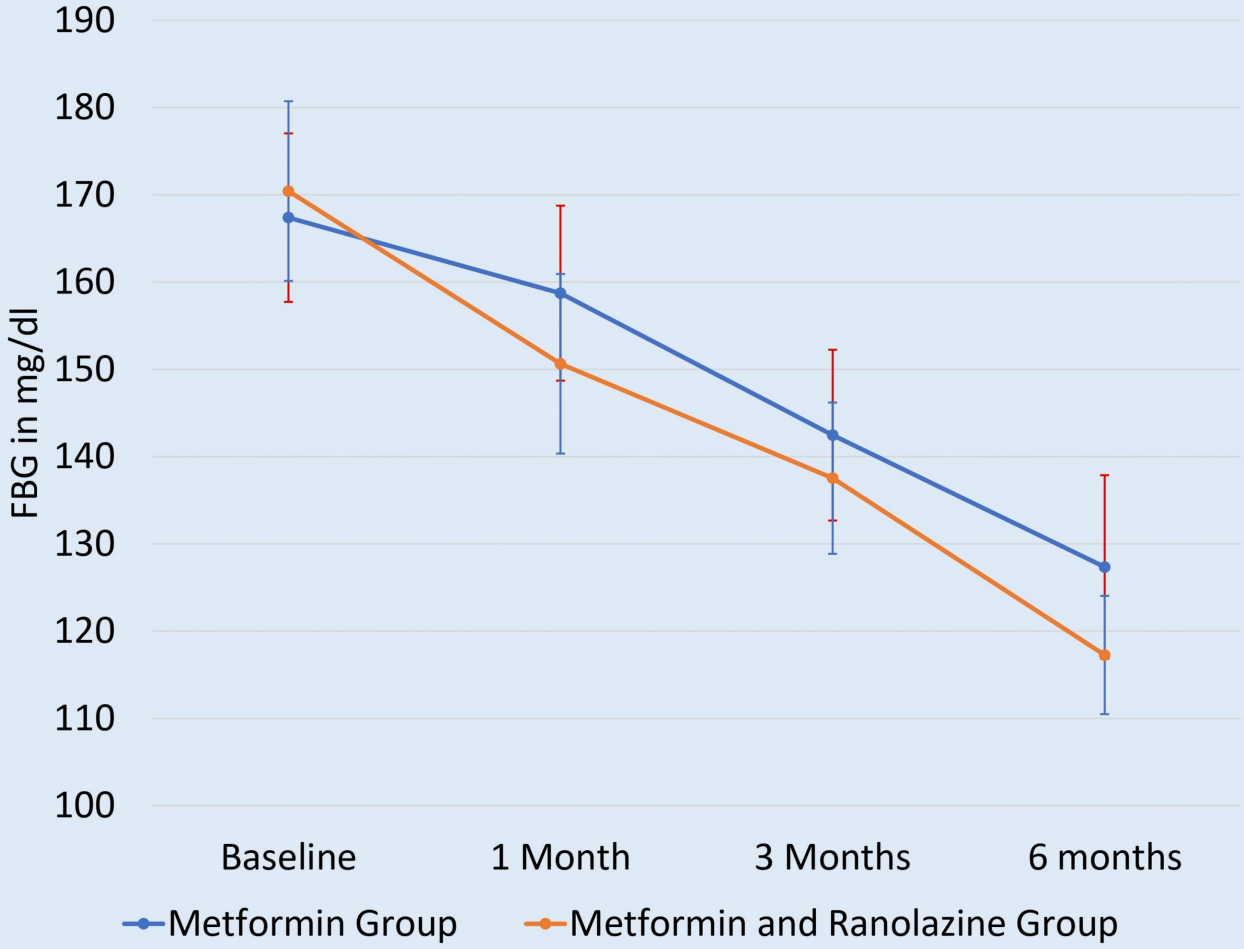
Mean FBG level at at different times of follow-up FBG: fasting blood glucose

At baseline, FBG levels were comparable (p = 0.761), indicating similar initial glycaemic profiles. However, at three months and six months, the combination therapy group demonstrated significantly lower FBG levels compared to the monotherapy group (p = 0.005 and p = 0.01, respectively), indicating enhanced fasting glycaemic control with the addition of ranolazine (Figure 2).

**Figure 3:**
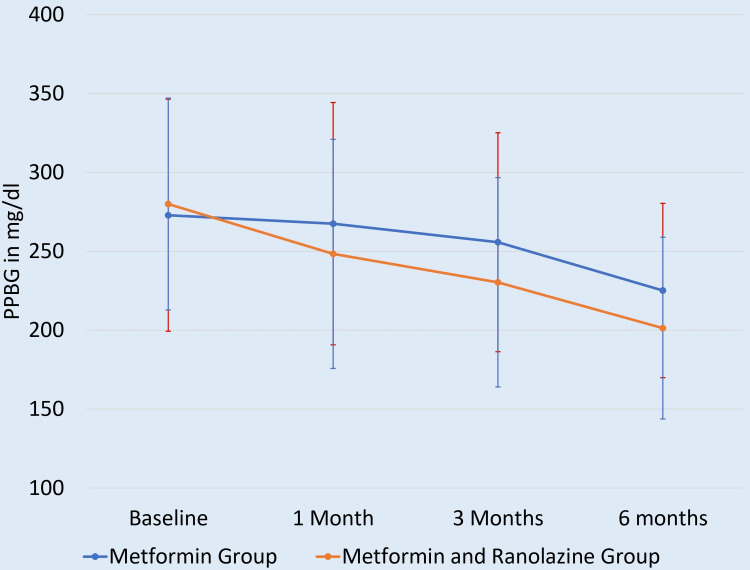
Mean PPBG level at different times of follow-up PPBG: post-prandial blood glucose level

At baseline, PPBG levels were comparable (p = 0.168), indicating comparable post-prandial glycaemic status. At three months, the combination group showed a trend toward lower PPBG levels, approaching statistical significance (p = 0.0501). By six months, the difference became statistically significant (p = 0.027), suggesting that the addition of ranolazine contributed to improved post-prandial glycaemic control over time (Figure 3).

The multivariable linear regression analysis identifies key predictors of change in HbA1c levels among patients receiving treatment. Statistically significant factors associated with increased HbA1c include longer period of diabetes (β = 0.015, p = 0.025), higher BMI (β = 0.014, p = 0.045), and elevated LDL levels (β = 0.010, p = 0.048). Conversely, higher baseline HbA1c is significantly associated with a greater reduction in follow-up HbA1c (β = -0.140, p = 0.002), suggesting regression toward the mean or enhanced responsiveness in poorly controlled individuals. Importantly, the treatment group receiving metformin plus ranolazine showed a significant HbA1c reduction compared to metformin alone (β = -0.121, p = 0.041), indicating potential additive glycaemic benefits. Other variables such as age, gender, systolic blood pressure, and LDL were not statistically significant predictors in this model (Table 4).

**Table 4:** Predictors of change in HbA1c; multivariable linear regression analysis * Statistically significant; CI: confidence interval; SBP: systolic blood pressure; LDL; low-density lipoprotein

Predictor variable	β coefficient	Standard error	95% CI	p-value
Age (per year)	-0.012	0.009	-0.030 to 0.006	0.19
Male gender (vs. female)	0.085	0.072	-0.057 to 0.227	0.24
Duration of diabetes (per year)	0.041	0.018	0.005 to 0.077	0.025*
BMI (per kg/m²)	0.022	0.015	-0.008 to 0.052	0.15
Baseline HbA1c (%)	-0.143	0.051	-0.244 to -0.042	0.006*
SBP (per mmHg)	0.004	0.003	-0.002 to 0.010	0.18
LDL (per mg/dl)	0.002	0.001	-0.000 to 0.004	0.048*
Treatment group (metformin + ranolazine vs. metformin)	-0.281	0.145	-0.567 to 0.006¹	0.054

## Discussion

The study investigated the effects of combining ranolazine with metformin in patients with T2DM and demonstrated significant improvements in glycaemic control compared to metformin monotherapy.

One of the most notable findings was the reduction in HbA1c levels in the metformin plus ranolazine group. At three months, the combination therapy group showed a statistically significant decrease in HbA1c (p = 0.022), which further improved by six months (p = 0.037). While the magnitude of reduction (-0.23% to -0.25%) was modest compared to some previous studies, such as Eckel et al. (2015), which reported a mean difference of -0.56%, it remains clinically meaningful [19]. Greiner et al. (2016) and Dampil et al. (2023) also documented similar HbA1c reductions (-0.28% to -0.7%), suggesting that ranolazine consistently contributes to better long-term glycaemic control [14,20]. Importantly, the study found no increased risk of hypoglycaemia, corroborating earlier findings that ranolazine is a safe adjunct without the drawbacks of excessive glucose-lowering.

The improvements in FBG were equally compelling. The combination group exhibited significantly lower FBG levels at both three months and six months (p = 0.005 & p = 0.01, respectively), reinforcing ranolazine's role in enhancing basal glycaemic regulation. These results mirror those of Chisholm et al. (2010), where ranolazine reduced FBG by 25.7 mg/dL in patients with poorly controlled diabetes and acute coronary syndrome [21]. The consistency across studies suggests that ranolazine's mechanism-possibly involving improved insulin sensitivity and reduced hepatic glucose output-plays a key role in stabilising fasting glucose levels.

Post-prandial glucose control also showed marked improvement with ranolazine. While the difference in post-prandial blood glucose (PPBG) at three months was borderline significant (p = 0.0501), it became clearly significant by six months (p = 0.027). This aligns with Eckel et al. (2015), where ranolazine monotherapy reduced two-hour post-prandial glucose by 19 mg/dL (p = 0.0008) [19]. The gradual but sustained improvement in PPBG suggests that ranolazine may modulate incretin effects or delay intestinal glucose absorption, though further mechanistic studies are needed to confirm this.

The study's findings fit well within the broader context of ranolazine research. For instance, Caminiti et al. (2016) demonstrated that ranolazine improved insulin resistance in non-diabetic patients with coronary heart disease, as measured by HOMA-IR [15]. While the current study did not assess insulin resistance directly, the FBG and HbA1c reductions suggest a similar metabolic benefit. Additionally, Nusca et al. (2021) highlighted ranolazine's ability to reduce glycaemic variability and improve endothelial function, which may explain its cardiovascular benefits in diabetic patients [22]. These complementary findings suggest that ranolazine's effects extend beyond glucose-lowering to include vascular protection, making it particularly valuable for patients with T2DM and cardiovascular disease.

Another critical comparison is with Kosiborod et al. (2013), which focused on ranolazine's antianginal effects in diabetic patients with chronic stable angina [23]. While the current study did not evaluate angina symptoms, the overlapping patient demographics (T2DM with potential CVD) suggest that ranolazine could offer dual cardiometabolic benefits, that is, improving glycaemic control while reducing ischemic symptoms. This is further supported by Chisholm et al. (2010), where ranolazine's glycaemic effects were most pronounced in patients with severe hyperglycaemia (HbA1c ≥8%), indicating that those with poorer baseline control may derive the greatest benefit [21].

The exact mechanisms behind ranolazine's glycaemic effects remain under investigation, but several hypotheses have been proposed. One possibility is pancreatic β-cell modulation. Ranolazine may enhance insulin secretion while simultaneously suppressing glucagon production. This is supported by findings from Dampil et al. (2023), who reported a reduction in fasting glucagon levels following ranolazine administration [20].

Another proposed mechanism involves improved insulin sensitivity. Caminiti et al. (2016) demonstrated significant reductions in HOMA-IR, suggesting that ranolazine may enhance peripheral insulin responsiveness [15]. Additionally, ranolazine may reduce hepatic glucose output. Inhibiting late sodium currents in hepatocytes could potentially suppress gluconeogenesis, thereby lowering endogenous glucose production. A further hypothesis is that ranolazine delays intestinal glucose absorption. Some evidence indicates that it may slow carbohydrate digestion, leading to improved post-prandial glycaemic control. This multifaceted action profile positions ranolazine as a potentially valuable adjunct therapy in clinical practice.

While the study provides valuable insights into the glycaemic benefits of combining ranolazine with metformin, several limitations must be acknowledged. The study population was restricted to single-centre patients on metformin monotherapy, excluding those on other antidiabetic regimens, which may limit applicability to broader T2DM populations.

## Conclusions

This study demonstrates that the addition of ranolazine to metformin therapy yields a statistically significant improvement in glycaemic control at three and six months, as evidenced by greater reductions in HbA1c, FBG, and PPBG compared to metformin alone. Although the absolute HbA1c change over six months showed a favourable trend in the combination group, it narrowly missed statistical significance. Baseline characteristics were well matched between groups, ensuring comparability. Multivariable regression analysis further confirmed that longer diabetes duration, higher LDL levels, and elevated baseline HbA1c were significant predictors of HbA1c change, while the treatment group showed a near-significant independent effect. Overall, ranolazine appears to offer incremental glycaemic benefits when added to metformin, warranting further investigation in larger, longer-term trials.
